# Bradykinesia-Akinesia Incoordination Test: Validating an Online Keyboard Test of Upper Limb Function

**DOI:** 10.1371/journal.pone.0096260

**Published:** 2014-04-29

**Authors:** Alastair J. Noyce, Anna Nagy, Shami Acharya, Shahrzad Hadavi, Jonathan P. Bestwick, Julian Fearnley, Andrew J. Lees, Gavin Giovannoni

**Affiliations:** 1 Reta Lila Weston Institute of Neurological Studies, UCL Institute of Neurology, London, United Kingdom; 2 Blizard Institute, Barts and the London School of Medicine and Dentistry, Queen Mary University of London, London, United Kingdom; 3 Wolfson Institute of Preventive Medicine, Barts and the London School of Medicine and Dentistry, Queen Mary University of London, London, United Kingdom; 4 The Royal London Hospital, Barts Health NHS Trust, Whitechapel, London, United Kingdom; Penn State College of Medicine, United States of America

## Abstract

**Background:**

The Bradykinesia Akinesia Incoordination (BRAIN) test is a computer keyboard-tapping task that was developed for use in assessing the effect of symptomatic treatment on motor function in Parkinson's disease (PD). An online version has now been designed for use in a wider clinical context and the research setting.

**Methods:**

Validation of the online BRAIN test was undertaken in 58 patients with Parkinson's disease (PD) and 93 age-matched, non-neurological controls. Kinesia scores (KS30, number of key taps in 30 seconds), akinesia times (AT30, mean dwell time on each key in milliseconds), incoordination scores (IS30, variance of travelling time between key presses) and dysmetria scores (DS30, accuracy of key presses) were compared between groups. These parameters were correlated against total motor scores and sub-scores from the Unified Parkinson's Disease Rating Scale (UPDRS).

**Results:**

Mean KS30, AT30 and IS30 were significantly different between PD patients and controls (p≤0.0001). Sensitivity for 85% specificity was 50% for KS30, 40% for AT30 and 29% for IS30. KS30, AT30 and IS30 correlated significantly with UPDRS total motor scores (r = −0.53, r = 0.27 and r = 0.28 respectively) and motor UPDRS sub-scores. The reliability of KS30, AT30 and DS30 was good on repeated testing.

**Conclusions:**

The BRAIN test is a reliable, convenient test of upper limb motor function that can be used routinely in the outpatient clinic, at home and in clinical trials. In addition, it can be used as an objective longitudinal measurement of emerging motor dysfunction for the prediction of PD in at-risk cohorts.

## Introduction

The Bradykinesia Akinesia Incoordination (BRAIN) test has been validated as a sensitive software tool for detecting signs of neurological disease, including Parkinson's disease (PD) and cerebellar dysfunction [1]. Based on the alternate finger tapping test, it has also been compared against the Unified Parkinson's Disease Rating Scale (UPDRS) and other PD severity scales [2]. Sequential finger tapping is part of the routine neurological examination for the detection of bradykinesia defined as ‘slowness of initiation of voluntary movement with progressive reduction in speed and amplitude of repetitive action’ [3]. Bradykinesia is tested as part of the motor section of the UPDRS, but severity of the sequence effect and the frequency of motor arrests are not specifically measured.

The traditional alternate finger tap test, in which the patient is observed tapping two mounted counters 15 cm apart as fast as possible has been used for many years to measure response to symptomatic drug treatment [4]. The BRAIN test replicates this test using a computer screen and keyboard and can provide additional information relating to the nature of the motor handicap in different neurological disorders. The user must alternately tap the ‘S’ and the ‘;’ keys as rapidly and as accurately as they can over a 30-second time period.

The original version of the BRAIN test was programmed to run in MS-DOS mode on an IBM-compatible personal computer [1]. We have developed a modified version that can run with all standard internet browsers. The patient or research volunteer completes the test online and the results are uploaded to a secure database for storage and analysis. Preliminary results on a small group of PD patients have already been reported [5]. In the present report, we have addressed the following issues:

1. The normal range of scores in healthy individuals and what influences these,
2. The difference in scores between PD patients and healthy individuals,
3. The reliability of the BRAIN test and its utility in repeat measurement.

## Methods

Ethics statement: All participants gave written informed consent via the BRAIN test website. The web-based consent form lists relevant consent statements and each statement has a check box. Participants must check each box before clicking the submit button on screen. The Queen Square Research Ethics Committee approved the entire study and the specific method of obtaining written consent.

Patients who fulfilled the Queen Square Brain Bank for Neurological Disorders criteria for the clinical diagnosis of PD and age-matched non-neurological controls were recruited from the outpatient department at the Royal London Hospital and the National Hospital for Neurology and Neurosurgery.^3^

Participants undertook the test seated at a desktop computer and keyboard. They followed on-screen test instructions and received no assistance during the test. Participants were allowed to choose which hand was tested first in acknowledgement of the fact that future use may frequently be unobserved. Each participant undertook two tests (one for each hand). Preliminary study established that a 30 second time period gave adequate information and would lead to greater compliance than testing for one minute [5]. Demographic data were recorded for all participants including gender, year of birth, level of education and self-reported hand dominance. For controls, additional co-morbidity information was recorded. For patients with PD, current medication, time of last dose of levodopa, number of years since diagnosis and Hoehn-Yahr stage were recorded. Patients with PD were examined using the motor section of the Movement Disorders Society (MDS) UPDRS by a trained clinician (AJN). The MDS-UPDRS includes a question about the clinical state of patients on medication. ‘On’ is the typical functional state when patients are receiving medication and have a good response and ‘Off’ is the typical functional state when patients have a poor response in spite of taking medications. These definitions of ‘On’ and ‘Off’ were recorded for each patient.

For reliability testing, 17 of the controls were asked to repeat the test multiple times for each hand. This had the secondary advantage of being able to investigate the possibility of a learning effect. Due to the tendency of motor features of PD to change in relation to medication and possible time of day, PD patients were not included in tests measuring reliability. However, six patients with PD and known motor fluctuations were invited to undertake the test on several occasions during the day, before and after medication in order to evaluate the BRAIN test in monitoring motor fluctuations.

The BRAIN test reports four variables calculated from raw data generated from key taps: kinesia score (KS30), the number of key taps in 30 seconds; akinesia time (AT30), the mean dwell time on each key in milliseconds (msec); dysmetria score (DS30), a weighted index using the number of incorrectly hit keys scored in a target fashion (1 point for the correct key, 2 points for immediately adjacent keys and 3 for other keys) then divided by the total number of key taps (i.e. if all keys are hit correctly, the score should be 1.0); and incoordination (or arrhythmia) score (IS30), the variance of the time interval in msec between keystrokes.

Descriptive statistics were calculated for all four variables. For continuous variables, means were reported if the data were normally distributed (assessed using the Shapiro-Wilks test) and medians were reported if not normally distributed. Within-group (PD or control) comparisons were performed using the paired t-test for normal distributed data or Wilcoxon-signed rank test for non-normally distributed data. Between-group comparisons were made using the unpaired t-test or Wilcoxon rank sum test. The sensitivity and specificity of test parameters separately and together were determined using logistic regression and receiver operated characteristic (ROC) curves. Associations between UPDRS and BRAIN test parameters were estimated using Spearman's rank correlation coefficient for non-normally distributed data. Coefficients of variation were calculated to determine reliability of test parameters in control subjects undertaking the test multiple times. The pre-determined significance level for all calculations was p = 0.05. All analyses were performed using Stata version 12 and GraphPad Prism version 6 for Mac.

## Results

There were 58 PD patients and 93 non-neurological controls included in the main analysis (for group characteristics see Table 1). One PD patient and one control subject were ambidextrous and were excluded from analyses that compared BRAIN test scores between the dominant and non-dominant hands. One PD patient tested in the clinic had incomplete UPDRS data and was excluded from those specific analyses.

**Table 1:** Demographic Information.

	PD	Controls
Number	58	93
Mean age (SD)	63.0 (10.6)	60.5 (13.1)
Gender		
- Male	37 (64%)	32 (34.4%)
- Female	21 (36%)	61 (65.6%)
Education		
- Primary	2 (4%)	4 (4%)
- Secondary	35 (60%)	46 (50%)
- Higher	7 (12%)	19 (20%)
- Further	14 (24%)	24 (26%)
Occupation		
- Professional	10 (17%)	26 (28%)
- Non-professional skilled	23 (40%)	37 (40%)
- Non-professional non-skilled	10 (17%)	22 (24%)
- Retired with no additional information	15 (26%)	8 (8%)
Handedness		
- Right	54 (93%)	81 (87%)
- Left	3 (5%)	11 (12%)
- Ambidextrous	1 (2%)	1 (1%)
Mean years since diagnosis (SD)	8.4 (6.6)	–
On/Off*		
- On	38 (66%)	–
- Off	20 (34%)	–
Levodopa		
- Yes	52 (90%)	–
- No	6 (10%)	–
Mean minutes since levodopa dose (SD)	186 (133)	–
Hoehn-Yahr stage		
- Stage 1	11 (19%)	–
- Stage 2	34 (59%)	–
- Stage 3	13 (22%)	–

PD, Parkinson's disease. SD, standard deviation.

*On/off in this table refers to the question in the MDS-UPDRS, which asks whether participants could feel the effects of medication at the time of examination. Note it does not indicate that all participants had motor fluctuations.

Associations of KS30, AT30, IS30 and DS30 with age, gender, education, occupation and co-morbidities were undertaken in controls (see Table 2). KS30 decreased by 0.66 points, AT30 increased by 1.35% and IS30 increased by 4.7% per year of age (all p<0.001). No significant correlation between DS30 and age was seen. Lower levels of education tended to give poorer scores for KS30 and AT30, and gave significantly poorer scores for IS30 and DS30. Analyses considering occupation showed that having a professional occupation gave significantly better KS30 scores, but there were no significant differences in the other parameters for different occupations. The presence of comorbidity did little to affect test results except for the finding that having any comorbidity worsened IS30 compared to those with no comorbidity and KS30 was non-significantly lower in those with depression. There was no significant effect of handedness and parameters were similar between males and females, except that females were significantly more accurate than males (improved DS30).

**Table 2:** Analysis of factors that influence KS30, AT30, IS30, DS30 in control subjects.

		KS30 (mean*)	p-value	AT30 (median*)	p-value	IS30 (median*)	p-value	DS30 (median*)	p-value
Mean age (SD)	60.45 (10.7)	r = −0.47	<0.0001	r = 0.31	0.0023	r = 0.34	0.0007	r = 0.16	0.12
Gender									
- Male	32	59.6	0.71	90.6	0.31	6887	0.73	1.062	0.03
- Female	61	60.7		99.2		6758		1.034	
Education									
- Primary	4	54.4	0.19	133.1	0.14	36913	0.03	1.232	0.02
- Secondary	46	58.0		99.9		7417		1.054	
- Higher	19	64.2		88.5		9496		1.038	
- Further	24	62.8		92.0		5446		1.027	
Occupation									
- Professional	26	65.7	0.05	92.0	0.48	5416	0.10	1.041	0.57
- Non-professional skilled	37	58.5		102.9		10645		1.042	
- Non-professional non-skilled	22	59.8		92.9		7402		1.063	
- Retired with no additional information	8	52.9		110.6		8939		1.030	
Co-morbidity									
- No additional	40	63.4	0.06	93.8	0.23	5072	0.02	1.034	0.37
- Affecting upper limbs including arthritis	7	55.3		125.2		13419		1.076	
- Depression	3	45.3		102.9		9310		1.000	
- Medical conditions	43	59.4		91.9		10645		1.044	
Handedness									
- Right	81	59.8	0.44**	99.2	0.65**	7093	0.46**	1.035	0.13**
- Left	11	63.1		91.6		4955		1.080	
- Ambidextrous	1	75		60.2		5384		1.109	

KS30, kinesia score; AT30, akinesia time; IS30, incoordination score; DS30, dysmetria score; CI, confidence interval; IQR, interquartile range; SD, standard deviation.

*Mean and medians given except for associations with age where correlation coefficient (r) is given. **p-values for comparisons between right and left handedness only.

**Figure 1:**
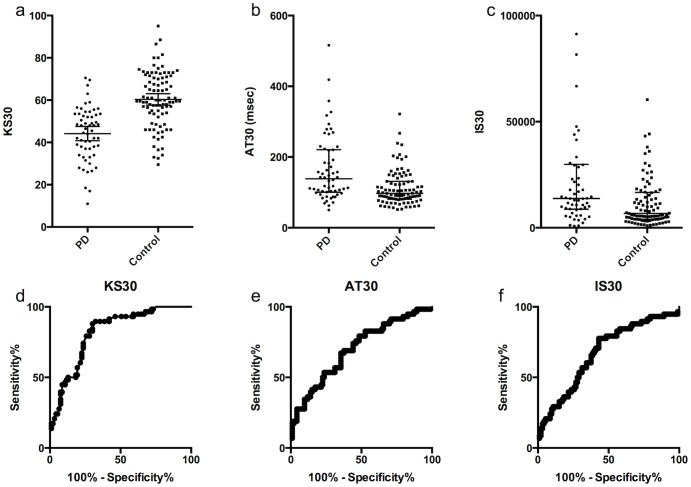
Comparison of KS30, AT30 and IS30 in patients with PD and controls (average of score from each hand). Distribution of KS30 (mean and standard deviation), (b) AT30 and (c) IS30 (medians and interquartile ranges). For IS30, 7 data points were out of the axis range. Receiver operating characteristic (ROC) curves for (d) KS30, (e) AT30 and (f) IS30.

When PD patients (n = 58) and controls (n = 93) were compared using averages of the scores from each hand; KS30, AT30 and IS30 discriminated between groups, but DS30 did not (see Table 3 and Figure 1). KS30 showed the best discrimination between PD and controls with sensitivities of 45%, 50% and 57% for specificities of 90%, 85%, and 80% respectively. Corresponding sensitivities were 31%, 40% and 43% for AT30 and 24%, 29% and 36% for IS30. The addition of AT30 or IS30 to KS30 did not improve discrimination compared with KS30 alone (assessed by multivariate logistic regression). When patients that were ‘On’ were excluded and patients that were ‘Off’ (n = 20) were compared to controls, the sensitivities for 90% specificity were 65%, 50% and 55% for KS30, AT30 and IS30 respectively. Subjects tested whilst ‘On’ had better KS30 scores than subjects who were tested whilst ‘Off’ (47.8 and 37.3 respectively, p = 0.002). IS30 scores were also significantly better in those that were ‘On’ (11682 and 29568 respectively, p = 0.007) and there was trend for improvement in AT30 (125.1 and 172.6 respectively, p = 0.27).

**Table 3:** Comparison of KS30, AT30, IS30, DS30 in all* PD patients versus controls.

		Mean KS30 (95% CI)	Median AT30 (IQR)	Median IS30 (IQR)	Median DS30 (IQR)
PD		44.2 (40.9, 47.5)	138.7 (100.5, 221.0)	13813 (8744, 29857)	1.042 (1.014, 1.147)
Controls		60.3 (57.6, 63.0)	97.3 (80.7, 131.2)	6758 (4030, 16664)	1.044 (1.013, 1.110)
p-value		<0.0001	<0.0001	0.0003	0.45
		KS30 Sensitivity	AT30 Sensitivity	IS30 Sensitivity	
		(cut-off)	(cut-off)	(cut-off)	
Specificity	90%	44.8%* (42.5)	31.0%* (183)	24.1%* (29954)	-
Specificity	85%	50.0% (46.0)	39.7% (156)	29.3% (23527)	-
Specificity	80%	56.9% (48.3)	43.1% (149)	36.2% (18527)	-

PD, Parkinson's disease; KS30, kinesia score; AT30, akinesia time; IS30, incoordination score; DS30, dysmetria score; CI, confidence interval; IQR, interquartile range.

*If analyses were limited to include only patients that were ‘Off’ (n = 20) the sensitivities (and cut-off) for 90% specificity for KS30, AT30 and IS30 were 65% (43), 50% (175) and 55% (29373) respectively.

Hands were compared in patients and controls (see Table 4). In both the dominant and non-dominant hands tests mean KS30 was significantly lower in PD patients than controls, and AT30 and IS30 were significantly higher. In patients and controls the dominant hand significantly out-performed the non-dominant hand for KS30 and AT30, but not for IS30 or DS30.

**Table 4:** Mean KS30 and median AT30 in all PD patients and controls according to hand.

	Mean KS30 (95% CI)			Median AT30 (IQR)			Median IS30 (IQR)			DS30 (IQR)		
	PD	Controls	p-value	PD	Controls	p-value	PD	Controls	p-value	PD	Controls	p-value
Dominant hand	46.5)	63.1	<0.0001	121	87	0.0002	10315	5969	0.0136	1.036	1.037	0.60
	(42.8,50.2	(60.1, 66.1)		(83,201)	(68,114)		(4830, 15562)	(2631, 14505)		(1.00,1.14)	(1.00, 1.08)	
Non-dominant hand	42.1	57.3	<0.0001	147	111	0.0002	12762	6162	0.0003	1.042	1.043	0.90
	(38.8, 45.5)	(54.6,59.9)		(112,232)	(87,155)		(5508, 33489)	(2966, 14040)		(1.00, 1.16)	(1.00, 1.11)	
p-value	<0.0001	<0.0001		<0.0001	<0.0001		0.74	0.78		0.91	0.12	

PD, Parkinson's disease; KS30, kinesia score; AT30, akinesia time; IS30, incoordination score; DS30, dysmetria score; CI, confidence interval; IQR, interquartile range.

NB. Ambidextrous PD patient excluded (first hand KS30 = 52, AT30 = 105; second hand KS30 = 36, AT30 = 175) and control excluded (first hand KS30 = 80, AT30 = 59; second hand KS30 = 81, AT30 = 60).

**Figure 2:**
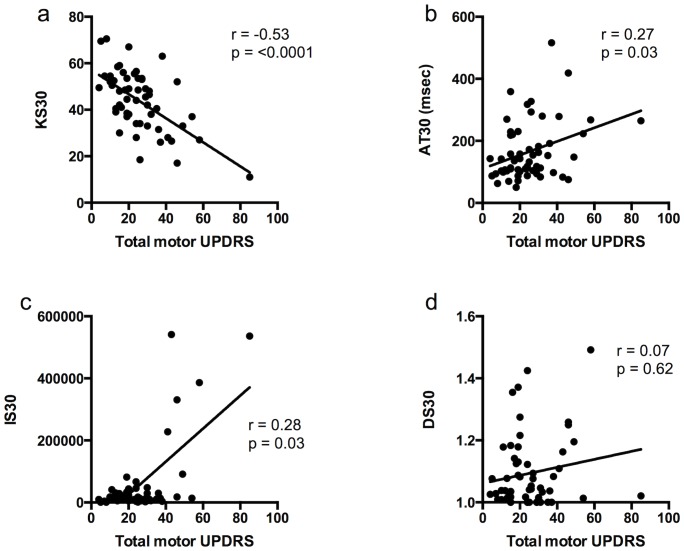
Correlation of (a) KS30, (b) AT30, (c) IS30 but not (d) DS30 with total motor UPDRS in patients with PD.

BRAIN test scores in PD patients only were compared to total motor UPDRS scores and sub-scores (see Figure 2) using averages of the scores from each hand. KS30 showed a moderate inverse correlation with total motor UPDRS (Spearman's r = −0.53, p<0.0001). AT30 and IS30 showed weak but significant positive correlations with total motor UPDRS (Spearman's r = 0.27, p = 0.03 and r = 0.28, p = 0.03 respectively). DS30 showed no correlation. Further correlations were undertaken with sub-sections of the UPDRS including upper limb tone, finger tapping, hand opening and closing, and pronation-supination (see Table 5).

**Table 5:** Correlations between test parameters and UPDRS sub-scores.

	Mean KS30 (95% CI)		Median AT30 (IQR)		Median IS30 (IQR)		DS30 (IQR)	
	Spearman's r	p-value	Spearman's r	p-value	Spearmanx's r	p-value	Spearman's r	p-value
Tone	−0.2085	0.0260	0.1656	0.0782	0.09565	0.3114	−0.02394	0.8004
Finger tapping	−0.4362	<0.0001	0.2007	0.0322	0.3289	0.0004	0.02581	0.7851
Hand movements	−0.5707	<0.0001	0.3538	0.0001	0.3941	<0.0001	0.05719	0.5456
Pronation supination	−0.3437	0.0002	0.3053	0.0010	0.2126	0.0231	−0.01070	0.9100

KS30, kinesia score; AT30, akinesia time; IS30, incoordination score; DS30, dysmetria score; CI, confidence interval; IQR, interquartile range.

In PD patients there was a difference of borderline significance with lower KS30 in the more affected hand when compared to the less affected hand (mean KS30 42.5 v 44.8, p = 0.053). There was no difference in AT30, IS30 and DS30 between the two hands (median AT30 134 v 128, p = 0.350; median IS30 12604 v 12048, p = 0.421, median DS30 1.034 v 1.051, p = 0.569). Duration of PD in years did not correlate with any of the four parameters (data not shown).

Seventeen of the controls repeated the BRAIN test five times for each hand to estimate the reliability of KS30, AT30, IS30 and DS30. The coefficient of variation for KS30 was 6.0%, for AT30 was 7.3%, and for DS30 was 3.4%. IS30 had a high coefficient of variation reflecting the fact that pauses in the test (even in control subjects) magnify the variance of travelling time significantly, decreasing the reliability of IS30 overall. There was a mild learning effect that saw KS30 increase by 1.2 taps per attempt (p = 0.002) but no learning effect for AT30 (decrease of 1.0 ms per attempt, p = 0.203) or for IS30 and DS30 (p values derived from multilevel mixed effects linear regression).

**Figure 3:**
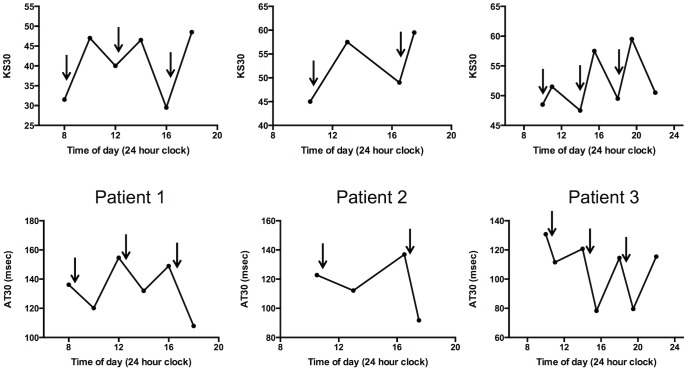
Examples of repeat tests in 3 PD patients with predictable motor fluctuation. Arrows denote times at which levodopa was taken.

Finally, using KS30 and AT30 the effects of medication were assessed in a small number of PD patients with predictable motor fluctuations (see Figure 3) and also in patients with unpredictable fluctuations (see Fure 4).

**Figure 4:**
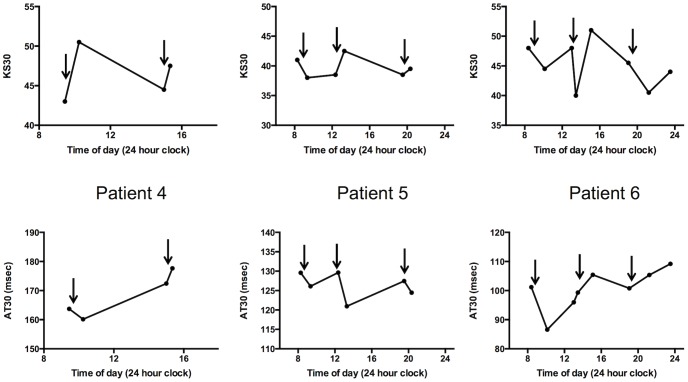
Further examples of repeat tests in 3 patients with predictable fluctuation (patient 4) and unpredictable motor fluctuation (patients 5 and 6). Arrows denote times at which levodopa was taken.

## Discussion

The BRAIN test has previously been shown to differentiate individuals with PD from healthy controls and also to correlate with PD severity measured by disease-specific rating scales [1], [2]. The results of these two studies, in which the original version of the test was performed on a single laptop computer under defined conditions, have been replicated here with this new online version of the test, allowing it to be administered without direct observation or investigator input. It can be accessed remotely from wherever there is an internet connection and a computer (laptop or desktop) keyboard.

In PD patients, KS30 correlated significantly with total motor UPDRS score, and limb specific sub-scores, as clinical indicators of motor disease severity. AT30 and IS30 also correlated significantly, albeit less strongly than KS30, with the total motor UPDRS score and some of the limb specific sub-scores.

Our data for PD and non-neurologic controls shows wide intra-group variability and yet the differences between KS30, AT30 and IS30 are highly significant and of clinically relevant magnitude, enabling cut-offs for sensitivity and specificity to be determined. We present results to optimize specificity, with resulting moderate to low sensitivity, thereby reducing the false-positive rate in acknowledgment that the test will often be performed remotely. Of course sensitivity and specificity operate on a continuum and much higher sensitivity can be achieved if the cut-offs are altered to accept a higher false positive rate.

Age clearly affected most of BRAIN test parameters and should be taken in consideration in future studies using the test. Education also influenced parameters perhaps reflecting an effect of computer literacy. However, this was not further reflected when examining occupation, with which strong relationships could not be found. This perhaps reflects widespread uptake of computers regardless of whether use is work-related or recreational. Patients that were tested in whilst ‘On’ performed better than those that were ‘Off’. When PD patients that were ‘Off’ were compared to controls the discriminative ability of KS30, AT30 and IS30 improved significantly. Comparison of most and least affected sides in PD patients only showed trends for poorer scores on the most affected side, perhaps reflecting bilateral involvement in 80% of the patient group (Hoehn and Yahr score 2 & 3).

Whilst not seemingly useful for differentiating PD from healthy controls, the dysmetria score (DS30) does provides a useful reference for judging whether tests have been completed properly, which is particularly valuable for remote testing. For example, the mean KS30 in controls is 60.3 and has a standard deviation of 13.1. Using three standard deviations as a cut-off, it is unlikely that an individual can exceed 100 alternate taps in 30 seconds without a dramatic loss of accuracy as reflected by the DS30. Occasionally very high scores have been seen in remote tests, such as those performed in the PREDICT-PD study (described further below), with perfect or near-perfect accuracy (e.g. KS30 of 200 and DS30 of 1.0) [6]. This suggests that the subject is using two hands to hit the keys and is not alternating between keys with a single hand, and such results should be excluded from analyses.

Some highly specialized tools have been developed that can accurately measure the specific motor deficit that occurs in PD and some have the capacity to differentiate the sequential tapping abnormalities in PD (true bradykinesia) from that seen in progressive supranuclear palsy and atypical tremors [7]–[9]. When compared to such tools, the BRAIN test appears fairly crude, but these specialist tests, whilst fulfilling a valuable role in research, are not currently applicable in routine clinical practice. Further study using the BRAIN test will now concentrate on whether a sequence effect can be demonstrated in patients that are ‘Off’ medication following a period of drug withdrawal and also studying greater numbers of patients with motor fluctuations in their ‘On’ and ‘Off’ phases, since the current study does not address these important aspects fully.

One criticism leveled at the BRAIN test during preliminary testing was that not all keyboards have identical characters, particularly outside the US and UK. Furthermore, the position of the ‘S’ and ‘;’ keys on the keyboard can vary between countries. These keys were originally chosen because they are 15 cm apart on a US/UK English standard desktop computer keyboard and most laptop computers. If one considers a standard keyboard divided in two halves by an imaginary line down the center, then these keys occupy a central position on their respective sides. In countries where US/UK English keyboards are not standard, the test can still be used with the keys that correspond to the position of ‘S’ and ‘;’ and has been implemented successfully by groups in Italy, Norway and the Netherlands (personal communications).

During testing we have found no evidence that use of different keyboards results in significant differences in results between subjects. However, use on tablet computers may be limited by the availability of the ‘;’ key and the different nature of touching a screen rather than pressing a key. As such we do not advise use of the BRAIN test on tablet computers or smart phones. In addition, use of sterile covers for keyboards in clinical settings may impair key presses. This might conceivably result in loss of accuracy (DS30) but is unlikely to affect the other three parameters (KS30, AT30 and IS30).

Undoubtedly the greatest value of the BRAIN test in established PD may come in the longitudinal monitoring of individual patients throughout the duration of their disease, including response to treatment and monitoring motor fluctuations. Repeat testing in controls suggests good reliability for three of the four parameters and only a minimal learning effect. In this analysis, control subjects repeated the tapping tests back-to-back and the fact that only a minimal learning effect was noted makes it unlikely that improvements due to learning would be seen were serial tests separated by days or weeks.

The BRAIN test has been successfully implemented as a motor assessment in our longitudinal online Parkinson’s risk study, PREDICT-PD, which began recruiting participants in April 2011 [6]. Individuals under follow-up in this study undertake the BRAIN test on an annual basis. Higher and lower risk groups were characterized on the basis of early non-motor symptoms for PD and recognized risk factors. When the 100 participants with the highest risk estimates where compared to 100 with lowest risk scores, significant differences in KS30 where found [6]. Other predictive and high-risk cohorts for PD have concentrated on early non-motor features and imaging abnormalities to ascribe risk of future conversion to PD [10], [11]. To our knowledge only one longitudinal study of PD risk has employed an objective computerized test. In the Honolulu Asia Ageing Study (HAAS) it was demonstrated that men in the slowest tertile of a reaction time test were significantly more likely to have Lewy body pathology at post mortem [12]. Postuma and colleagues have followed up a large cohort of patients with REM sleep behavior disorder (RBD), which is a strong risk factor for PD [13]. They demonstrated that motor deterioration could be measured for approximately 4-8 years across a number of motor domains (including alternate finger tapping) prior to the diagnosis of PD. The premotor period has been estimated to last between 5–15 years prior to the diagnosis of PD [14]. We prefer the term pre-diagnostic PD in this context; given the stringent motor criteria that must be met for a clinical diagnosis (including demonstration of a sequence effect), it seems likely that subtle motor dysfunction must be present at an earlier stage [15], [16].

The online BRAIN test represents a simple, validated, objective tool to longitudinally monitor motor function not only in established PD but also in studies seeking to identify those at high risk of future PD. The BRAIN test can be accessed at www.braintaptest.com. Tokens for individual use can be requested by clinicians and researchers via the website.
