# Correction: Bradykinesia-Akinesia Incoordination Test: Validating an Online Keyboard Test of Upper Limb Function

**DOI:** 10.1371/journal.pone.0105488

**Published:** 2014-08-06

**Authors:** The PLOS ONE Staff

There is an error in the funding statement for this article. Please refer to the correct funding statement below:

AJ Noyce declares the following: consultancy, Élan/Prothena Pharmaceuticals; Grants, Parkinson’s UK Innovation Grant (reference number K-1006), Parkinson’s UK Career Development Award (F-1201), and National Institute of Health Research Academic Clinical Fellowship. Funding came from departmental resources. The funders had no role in study design, data collection and analysis, decision to publish, or preparation of the manuscript.
